# Gallium-68 Citrate PET/CT for Diagnosis and Treatment Response Assessment of Infections—Prospective Study

**DOI:** 10.1055/s-0045-1807256

**Published:** 2025-04-01

**Authors:** Hanna Elizabeth Johnson, Saumya Sara Sunny, David Mathew, Regi Oommen, Nylla Shanthly, Priscilla Rupali, Regi Thomas, Rajan Sundaresan, Sumant Samuel, Anil Oommen, Venkatesh Krishnan, Julie Hephzibah

**Affiliations:** 1 Department of Nuclear Medicine, Christian Medical College and Hospital, Christian Medical College, Vellore, Tamil Nadu, India; 2 Department of Infectious Diseases, Christian Medical College and Hospital, Vellore, Tamil Nadu, India; 3 Department of Otorhinolaryngology, Christian Medical College and Hospital, Vellore, Tamil Nadu, India; 4 Department of ENT-1, Head & Neck Skull Base Surgery Unit, Christian Medical College, Vellore, Tamil Nadu, India; 5 Department of Orthopedics, Christian Medical College and Hospital, Vellore, Tamil Nadu, India; 6 Department of Spinal Disorder Surgery, Christian Medical College and Hospital, Vellore, Tamil Nadu, India

**Keywords:** Ga-68 citrate, imaging, infection, osteomyelitis, PET/CT

## Abstract

**Objective:**

This article aims to assess the presence of skeletal and soft tissue infections before or after treatment and to assess treatment response in Ga-68 citrate positron emission tomography/computed tomography (PET/CT) scan positive patients.

**Materials and Methods:**

A prospective study was conducted for 43 patients. The eligibility criteria included those patients clinically suspected of infections who underwent a Ga-68 citrate PET/CT. Exclusion criteria were pregnancy and lactation. Patients with suspicion of infection or treatment failure underwent a Ga-68 citrate PET/CT between January 2020 and November 2021. Among these, eight patients underwent a follow-up scan posttreatment to assess their treatment response. The Institutional Review Board (IRB No.12511) approved the study.

**Results:**

Forty-three patients underwent a diagnostic Ga-68 citrate PET/CT scan. The scan interpretation was based on visual comparison of uptake of Ga-68 citrate in the region of interest, which was compared with the normal side/adjacent soft tissue/blood pool. The semiquantitative parameter maximum standardized uptake value was retrospectively analyzed as well. PET/CT findings were correlated with tissue diagnosis, clinical symptoms, biochemical parameters like C-reactive protein (CRP), erythrocyte sedimentation rate, and total leukocyte count, and other imaging modalities with a statistically significant association with CRP ( *p* = 0.001). Tissue diagnosis was considered the gold standard and out of the 43 patients included in the study, 27 had a tissue diagnosis. Sensitivity, specificity, negative predictive value, positive predictive value, and accuracy were calculated at 100, 87.5, 100, 95, and 96.3%, respectively.

**Conclusion:**

Ga-68 citrate is a promising tool to assess the presence of bone and soft tissue infections before or after treatment.

## Introduction

For decades, nuclear medicine has been at the forefront of infection imaging and has explored the utility of Ga-67 citrate for diagnosing various infections. Ga-67 citrate has been well-known for decades as the imaging agent for detecting infections. 1 It has been used for the diagnosis of various clinical conditions such as pyrexia of unknown origin, autoimmune-related inflammations, sarcoidosis, pancreatitis, idiopathic pulmonary fibrosis, osteomyelitis, pulmonary Wegener's granulomatosis, and chronic bronchial asthma. 2 However, due to its various drawbacks, it has slowly become unpopular. Compared with Ga-68, Ga-67 has a much longer half-life of 78 hours and emits high-energy gamma radiations ranging from 92 to 300 KeV. 1 3 4 Due to slow uptake, delayed imaging of up to 72 hours is required. 2 The high energy of the radiation gives it unfavorable imaging characteristics and high radiation exposure to the patients. Ga-67 imaging uses a gamma camera, and planar imaging often misses deep-seated lesions and requires supplementation by acquisition with single-photon emission tomography. The effective dose of Ga-68 per unit of administered activity is 2.6 × 10–2 mSv/MBq, while Ga-67 is 1.1 × 10–1 mSv/MBq. 5 Ga-67 requires a cyclotron for production, making the tracer much more expensive than Ga-68, produced via an onsite Ge-68/Ga-68 generator. Ga-68 is a positron emitter, and images are acquired via positron emission tomography/computed tomography (PET/CT) acquisition, 3 which gives the advantage of anatomical imaging. 6 A shorter half-life, lower radiation dose, better imaging characteristics, and lower cost make Ga-68 citrate a better choice for an imaging agent. 7 8 Several studies have investigated the ability of Ga-68 citrate to differentiate between infection and aseptic inflammation and assess treatment response. 9 10

Ga-68 citrate is a radiopharmaceutical explored in the recent past and showed promising results. Ga-68 citrate follows a similar mechanism of uptake as Ga-67 citrate. 11 Areas of infection have an abundance of leukocytes. Gallium in plasma is bound tightly to iron transport protein transferrin (TF) and enters the cell through the TF receptor, thus accumulating in infectious foci. Macrophages also have high expression of TF receptors. There is increased capillary permeability at sites of infections, and gallium leaks from the vascular epithelium at these sites. It is also taken up directly by the pathogen itself. It then binds to lactoferrin (LF), which is present in the leukocytes. The gallium-bound LF then binds to the activated macrophages present at the site of inflammation. Pathogens causing infection produce siderophores due to little free iron present in tissues. These siderophores have a high affinity for iron and gallium due to their similarity in their structure and properties. The gallium–siderophore complex retains itself within the cell after direct entry. It has found recent interest amongst nuclear medicine professionals due to the ease of production, the lower cost, lower radiation exposure to the patient, and better patient compliance due to shorter scan time. Conventional imaging, 12 like CT, X-rays, and ultrasonography (USG), may miss the early stages of infection when apparent structural abnormalities are lacking. Magnetic resonance imaging (MRI) is very sensitive and has the advantage of detailed anatomy but is of limited value in patients with metallic implants and the presence of postoperative edema or aseptic inflammation. 12 F-18 fluorodeoxyglucose (FDG) can be used for infection imaging but lacks specificity due to its mechanism of uptake in both inflammation and infections; it also has the disadvantage of cyclotron production. FDG is taken up by any metabolically active cell as it is a glucose analog and accumulates in the cell after phosphorylation. White blood cells (WBCs) imaging is sensitive, but the procedure is long and cumbersome 13 with a higher radiation exposure 14 to radiation personnel involved in radiopharmaceutical preparation. WBC imaging has limited value in the setting of neutropenia, a limitation which can be overcome using Ga-68 citrate. Ga-68 citrate is a generator-produced radiopharmaceutical and helps differentiate infection from aseptic inflammation. 15

In our study, we looked at the incremental value of Ga-68 citrate in the diagnosis of infection. Our study comprised the following patient groups: (1) skull base osteomyelitis (SBO), (2) long bone infections, (3) prosthesis-related infections, (4) end-of-treatment assessment for spinal tuberculosis, and (5) soft tissue infections.

## Material and Methods

### Study Subjects

A prospective study was conducted for 43 patients. The eligibility criteria included those patients clinically suspected of infections who underwent a Ga-68 citrate PET/CT. Exclusion criteria were pregnancy and lactation. Patients with suspicion of infection or treatment failure underwent a Ga-68 citrate PET/CT between January 2020 and November 2021. Among these, eight patients underwent a follow-up scan posttreatment to assess their treatment response. The Institutional Review Board (IRB No.12511) approved the study.

### [68Ga] Citrate PET/CT Imaging

Ga-68 was eluted from the Ge-68/Ga-68 generator with hydrogen chloride after labeling with buffer citrate solution for 10 minutes and passing through the Waters cartridge. Each patient received an intravenous dose of 3 to 5 mCi/111 to 185 MBq of Ga-68 citrate. Ga-68 citrate was administered intravenously with an uptake time of 60 minutes. A CT scan with contrast when needed was performed first with the following parameters: tube current of 110 mAs, tube voltage of 130 kV, and slice thickness of 3 to 5 mm. Subsequently, PET images were obtained through the same region, with each bed position lasting 2 minutes and a total of three beds. Processing of the images was performed using iterative reconstruction followed by a PET scan for the area of interest 1 hour post-intravenous administration of Ga-68 citrate, following which PET imaging for the area of interest was performed. The interpretation was based on a visual assessment of Ga-68 citrate tracer uptake in the region that is suspected of infection, and the tracer uptake in the scan was correlated with clinical, biochemical, microbiological, and other radiological parameters.

### Imaging Interpretation

Visual interpretation of images was performed based on uptake of the tracer in the area of suspicion. The visual comparison was made with the normal side/adjacent soft tissue/blood pool. Image interpretation was performed by at least two experienced nuclear medicine physicians.

CT images were viewed and interpreted in detail by an experienced radiologist for anatomical correlation. Almost all patients with diagnosis of SBO additionally underwent MRI as well.

Semiquantitative analysis using maximum standardized uptake value (SUVmax) was performed retrospectively, due to which data of 12 patients could not be retrieved. The SUVmax was compared with the opposite normal side soft tissue/bone or the blood pool uptake.

### Histopathological Examination

Among the 43 patients, 27 patients underwent tissue diagnosis in the form of biopsy or pus culture.

### Statistical Analysis

For continuous data, the descriptive statistics mean and standard deviation were reported. All categorical variables were represented as numbers and percentages. The chi-square and Fisher's exact tests (less cell count) were used to find the association between categorical variables. All tests were two-sided at *α* = 0.05 level of significance. All analyses were done using Statistical Package for Social Sciences (SPSS) software Version 21.0 (IBM Corp, Armonk, New York, United States).

## Results

A total of 43 patients with suspicion of infection or treatment failure who met the inclusion criteria were included in the study. Among these, eight patients had a follow-up scan posttreatment to assess treatment response. The median duration of follow-up was 3 to 6 months. The mean age of males (72%) was 54.8 years and that of females (28%) was 51 years (concise patient profile is provided in Table 1 ).

**Table 1:** Concise patient profile

Total number, *N* = 43	Male; *n* = 31	Female; *n* = 12
Mean age	Male: 54.8 y	Female: 51 y
Diagnosis	Skull base osteomyelitis, *n* = 18 Infected prosthesis, *n* = 12 Spinal tuberculosis, *n* = 1 Soft tissue infections, *n* = 5	
Treatment naive	Yes, *n* = 18	No, *n* = 25
Tissue diagnosis (biopsy/culture)	Yes, *n* = 27	No, *n* = 16

### Patient Profile

Among the 43 patient patients (clinical details in Table 2 ), the provisional/proven diagnosis included 18 patients with SBO, 7 patients with long bone osteomyelitis, 12 patients with an infected prosthesis, 1 patient with a posttreatment assessment of spinal tuberculosis, and 5 patients with soft tissue infection that included: right pyelonephritis with prostatic abscess, to evaluate surgical bypass graft site infection of the left femoral-posterior tibial artery, to evaluate infective etiology for an abdominal aortic aneurysm, to evaluate infective etiology for saccular aneurysm of the abdominal aorta below the superior mesenteric artery, and to differentiate between an infective versus hemorrhagic cysts in a patient with autosomal dominant polycystic kidney disease (ADPKD) with persistent fever.

**Table 2:** Clinical details of patients

Sl. no.	Diagnosis	Pretreated (surgical/medical)	Ga-68 citrate PET/CT (based on visual assessment)	SUVmax (ROI)/SUVmax (blood pool or normal side)	Correlating parameters with Ga-68 citrate PET/CT findings	Final diagnosis
1.	? SBO	No treatment	Positive	6.21 / 4.09 (normal side)	Biopsy and pus culture: positive	True positive
2.	? Left knee infected implant, k/c/o rheumatoid arthritis	No treatment	Negative	3.32 / 5.12 (blood pool)	Pus culture: negative	True negative
3.	Right SBO, DM	Surgery	Positive	9.2 / 9.02 (opposite side)	Biopsy and pus culture: positive	True positive
4.	Left SBO with cranial nerve palsies. Left vagal and hypoglossal nerve palsy, DM, HTN	Surgery	Positive	6.83 / 5.32 (normal side)	Biopsy and pus culture: positive	True positive
5.	Prostatic abscess, right pyelonephritis with prostatic abscess, DM, diabetic nephropathy, diabetic neuropathy, HTN	No treatment	Positive	17.98 (prostate) / 10.54 (blood pool)	Biopsy and pus culture: positive	True positive
6.	? SBO, right lower motor neuron palsy, DM	No treatment	Positive	–	Biopsy and pus culture: positive	True positive
7.	Left SBO with CN palsies, DM. HTN	Surgery	Positive	7.44 / 5.58 (normal side)	Biopsy and pus culture: positive	True positive
8.	Central SBO	Medical	Negative	2.3 / 8.50 (blood pool)	Clinical doing well and continued to clinically do well at 1-month follow-up	Negative a
9.	Right femur fracture, post-intermedullary nailing 1 year ago	Medical	Negative	8.39 / 7.72 (blood pool)	No other investigations / blood tests, as the patient was lost to follow-up	Inconclusive
10.	? SBO, DM	No treatment	Positive	7.69 / 6.93 (blood pool)	Biopsy and pus culture: positive	True positive
11.	Left chronic hip arthritis, to rule out infection / secondary to spondyloarthritis	Medical	Positive	6.24 / 5.68 (blood pool)	ESR and CRP: high	Positive a
12.	Surgical graft site infection 1 month prior (MRSA) - post left femoral-posterior tibial artery bypass graft, to rule out residual / recurrent infection	Medical	Negative	2.35 / 6.0 (blood pool)	Pus culture: negative	True negative
13.	Right femur shaft and proximal femur nonunion implant in situ, to look for infectious cause	Surgery	Negative	2.07 / 7.77 (blood pool)	Pus culture: negative	True negative
14.	Right humerus, chronic OM, DM, posttreatment	Medical	Negative	–	CRP: low Clinically has full range of movement, no bony tenderness, with occasional pain	Negative a
15.	Nonhealing ulcer, right forearm. History of both bone fracture post-RTA, ? implant-related osteomyelitis	Medical	Negative	–	Pus culture, biopsy: negative	True negative
16.	L4–5 infective tubercular spondylodiscitis	Medical	Negative	–	MRI: negative CRP: low	Negative a
17.	Abdominal aortic aneurysm ? infective cause	No treatment	Negative	–	Pus culture: negative	True negative
18.	SBO with TM joint involvement, DM, HTN, CKD 4	Surgery	Positive	13.65 / 3.34 (normal side)	Biopsy: positive	True positive
19.	RTA 2004, post-IM nail right femur, implant exit in 2005 due to infection. H/o debridement surgeries in 2007, 2009, and 2013	Medical	Positive	5.03/1.44 (normal side)	ESR and CRP: high - Clinical: ROM restricted and pain and discharging pus	Positive a
20.	Saccular aneurysm of abdominal aorta below SMA on CT. To rule out infective etiology. CKD 4	No treatment	Negative	–	Biopsy: negative	True negative
21.	Chronic OM right ankle with implant in situ, HIV stage 3	Medical	Positive	3.52/0.3 (normal side)	Biopsy and pus culture: positive	True positive
22.	Left femur, acute on chronic OM	Surgery	Positive	6.73 / 2.79 (normal side)	Biopsy and pus culture positive	True positive
23.	Left SBO, DM, HTN, CKD on HD	No recent treatment	Positive	5.3 / 4.46 (normal side)	MRI +	Positive a
24.	Right distal femur infective nonunion Diabetes mellitus-II	Surgery	Positive	5.47 / 6.34 (blood pool)	Biopsy and pus culture: positive	True positive
25.	Left forearm both bone infected nonunion and implant failure status post-left forearm both bone ORIF-2017	No recent treatment	Positive	3.38 / 2.06 (normal side)	Pus culture: positive	True positive
26.	Post-bilateral knee replacement with chronic right knee pain -to look for focus of infection	No recent treatment	Positive	7.28 / 1.09 (normal side)	CRP, ESR: high	Positive a
27.	Chronic SBO, diabetes mellitus, chronic kidney disease	Surgery	Positive	–	Biopsy and pus culture: positive	True positive
28.	ADPKD and persistent fever, to rule out infection or hemorrhage	No treatment	Positive	–	Biopsy and pus culture: negative	False positive
29.	SBO, DM	Surgery	Positive	–	Biopsy positive	True positive
30.	Chronic osteomyelitis of right femur, to look for acute on chronic OM	Medical	Negative	–	CRP, TLC, and ESR: normal	Negative a
31.	Central SBO and left temporomandibular joint involvement - secondary to left malignant otitis externa DM, HTN	Surgery	Positive	–	Biopsy positive	True positive
32.	Central SBO with left 9, 10, 11, 12 cranial palsies HTN	No treatment	Positive	–	Biopsy and pus culture positive	True positive
33.	Right Austin Moore prosthesis with? loosening / infection	No recent treatment	Negative	3.48 / 5.16 (blood pool)	CRP and ESR: low	Positive a
34.	Suspected skull base osteomyelitis	No treatment	Positive	12.26 / 4.51 (blood pool)	Clinical: Left ear pain, blood-stained discharge, and hearing loss for 3 months	Positive a
35.	Right knee prosthetic joint infection	Medical	Positive	6.49 / 2.00 (normal side)	ESR: high CRP: normal	Positive a
36.	Recurrent skull base osteomyelitis	Medical	Positive	6.39 / 1.79 (normal side)	CRP: high, MRI: positive	Positive a
37.	Lower backache with right knee pain, post-TKR done elsewhere. To look for infection.	No treatment	Negative	1.60 / 4.0 (blood pool)	CRP and TLC: low	Negative a
38.	Malignant otitis externa with suspected skull base osteomyelitis	No treatment	Negative	3.18/3.80 (normal side)	TLC: low Clinical symptoms improved	Negative a
39.	Periprosthetic fracture, post-right THR. To rule out infection	No recent treatment	Positive	6.68 / 5.62 (normal side) and 6.92 blood pool	CRP, ESR, TLC: high	Positive a
40.	Post-bilateral TKR. ? Right knee infection	No recent treatment	Positive	9.25 / 5.05 blood pool	Biopsy positive	True positive
41.	Skull base osteomyelitis	Surgery	Positive	10.12 / 5.28 (normal side)	Biopsy and pus culture positive	True positive
42.	Skull base osteomyelitis	Surgery	Positive	5.63 / 3.03 (normal side)	Biopsy positive	True positive
43.	Periprosthetic joint infection of left hip, status post-debridement, and implant removal, c/o pain	Surgery	Negative	2.65 / 8.58 (blood pool)	Biopsy negative	True negative

Abbreviations: ADPKD, autosomal dominant polycystic kidney disease; CN, cranial nerve; C/o, complaints of; CKD, chronic kidney disease; CRP, C-reactive protein; DM, diabetes mellitus type 2; ESR, erythrocyte sedimentation rate; H/o, history of; HD, hemodialysis; HIV, human immunodeficiency virus; HTN, hypertension; IM, intramedullary; MRI, magnetic resonance imaging; MRSA, methicillin-resistant *Staphylococcus aureus* ; OM, osteomyelitis; ORIF, open reduction and internal fixation; PET/CT, positron emission tomography/computed tomography; ROI, region of interest; ROM, range of movement; RTA, road traffic accident; SBO, skull base osteomyelitis; SMA, superior mesenteric artery; SUVmax, maximum standardized uptake value; THR, total hip replacement; TKR, total knee replacement; TLC, total leukocyte count.

a Positive or negative based on combination parameters, without a gold standard.

### Ga-68 Citrate PET/CT and Other Infective Parameter Assessment

Results were concluded based on an association between the Ga-68 citrate PET/CT scan findings and other parameters such as biopsy, pus culture, biochemical infective markers, MRI findings, and clinical outcome. Inflammatory markers such as C-reactive protein (CRP), erythrocyte sedimentation rate (ESR), and total leukocyte count (TLC) were also evaluated.

Tissue diagnosis was considered the gold standard and 27/43 had a tissue diagnosis. Sensitivity, specificity, negative predictive value, positive predictive value, and accuracy were calculated at 100, 87.5, 100, 95, and 96.3%, respectively. Out of the 16 patients who did not have a tissue diagnosis, Ga-68 citrate PET/CT scan interpretation was based on visual assessment, clinical symptoms, and correlation with biochemical markers like CRP, ESR, TLC, and other imaging modalities.

Positive Ga-68 citrate scan findings for infection had a statistically significant association with inflammatory markers CRP ( *p* = 0.001), which was high in 75% ( *n* = 25/33) patients. Tissue diagnosis with biopsy and pus culture in 85% ( *n* = 17/20) and 56% ( *n* = 14/25) patients, respectively, were suggestive of infection and showed statistically significant association with Ga-68 citrate scan findings with a *p* -value of 0.016 for biopsy and *p* -value of 0.009 for pus culture. Only 16 (predominantly consisting of SBO patients) out of 43 patients underwent an MRI scan, 15 of whom had findings positive for infection on both MRI and Ga-68 citrate PET/CT. But statistically significant association ( *p* = 0.06) could not be evaluated due to the small number of patients that underwent an MRI scan.

Out of the 43 patients included in the study, 18 patients were treatment naive, and 25 patients had received treatment prior to imaging. Among the 18 treatment-naive patients, 11 patients had biopsy correlation, of which, 7 were true positives, 3 were true negatives, and 1 patient was false positive. Among the 25 pretreated patients, 16 had tissue biopsy, of which 12 were true positives and 4 were true negatives. It is noteworthy that there were no false negatives among the patients who had a biopsy.

**Fig. 1:**
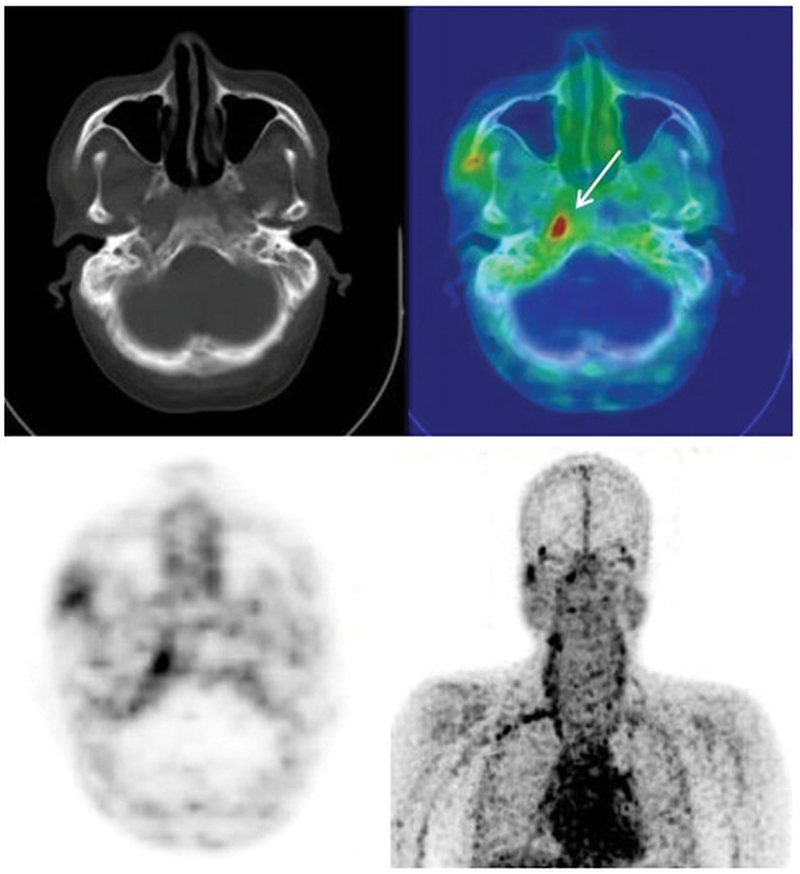
A 46-year-old lady with history of left lateral and central skull base osteomyelitis (SBO), for assessment, Ga-68 citrate positron emission tomography/computed tomography (PET/CT) done showed citrate avid significant enhancing soft tissue thickening in the left carotid space completely encasing the left internal carotid artery, involving the left petrous apex and the left half of clivus.

**Fig. 2:**
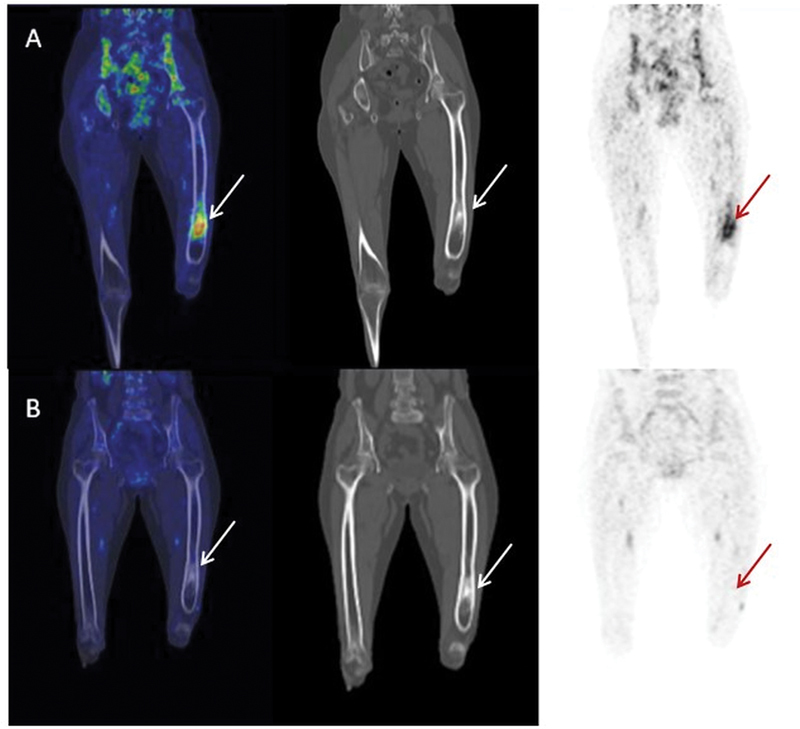
A 60-year-old lady with history of left femur osteomyelitis with persistent pain. ( **A** ) Ga-68 citrate positron emission tomography/computed tomography (PET/CT) done showed increased citrate uptake in the left distal femur with corresponding CT showing diffuse cortical thickening and remodeling of the shaft of the left femur, multiple small intramedullary calcific foci, and mild focal replacement and medullary fat with soft tissue in the lower third of femoral diaphysis; features suggestive of an acute osteomyelitis. Pus culture and biopsy was also suggestive of acute osteomyelitis. Patient underwent left femur debridement with cortical window and curettage and a follow-up was done as shown in ( **B** ): Ga-68 citrate PET/CT showed resolution of previously noted citrate avidity with CT images showing postoperative cortical defect in the left distal femur with cortical thickening and ill-defined lucencies seen in the intramedullary region.

Our study evaluated 18 (41%) patients with suspected SBO ( Fig. 1 ) and 7 (16%) patients with long bone osteomyelitis ( Fig. 2 ). Out of the 18 patients with SBO, 16 had a Ga-68 citrate PET/CT scan that was positive for infection. Of these 16, 13 patients had tissue diagnosis suggestive of infection, of which 9 patients had both biopsy and pus cultures positive for infection, 4 patients had biopsy suggestive of osteomyelitis, 2 patients had an MRI with findings suggestive of osteomyelitis, and 1 patient who clinically presented with left ear pain and mucopurulent blood-stained discharge had a positive Ga-68 citrate scan.

Six patients underwent Ga-68 citrate PET/CT scan for diagnosis of suspected long bone osteomyelitis, and one patient with chronic hip arthritis to look for any focus of infection. Four had a positive scan, of which three had a positive biopsy and pus culture, confirming the scan findings, and one had high inflammatory markers. Three had a negative Ga-68 citrate PET/CT scan, of which one patient had a negative pus culture and biopsy, and the other two patients had a normal CRP, which, as per our study, showed significant association with Ga-68 citrate PET/CT scan findings.

Twelve patients were evaluated for suspected prosthesis-related infections, of which 7 (58%) were joint prostheses and 5 (42%) were long bone prostheses. Of the 12 patients, 7 had a Ga-68 citrate scan findings suggestive of infection and 5 patients had tissue diagnosis suggestive of infection (2 patients had a positive biopsy and 3 had a positive pus culture), and good association was found with raised inflammatory markers (6/7 patients had raised ESR and CRP levels). Among the patients with negative Ga-68 citrate PET/CT ( *n* = 5), two were true negative with negative pus culture growth and three patients who did not have tissue confirmation had normalized CRP and TLC levels.

**Fig. 3:**
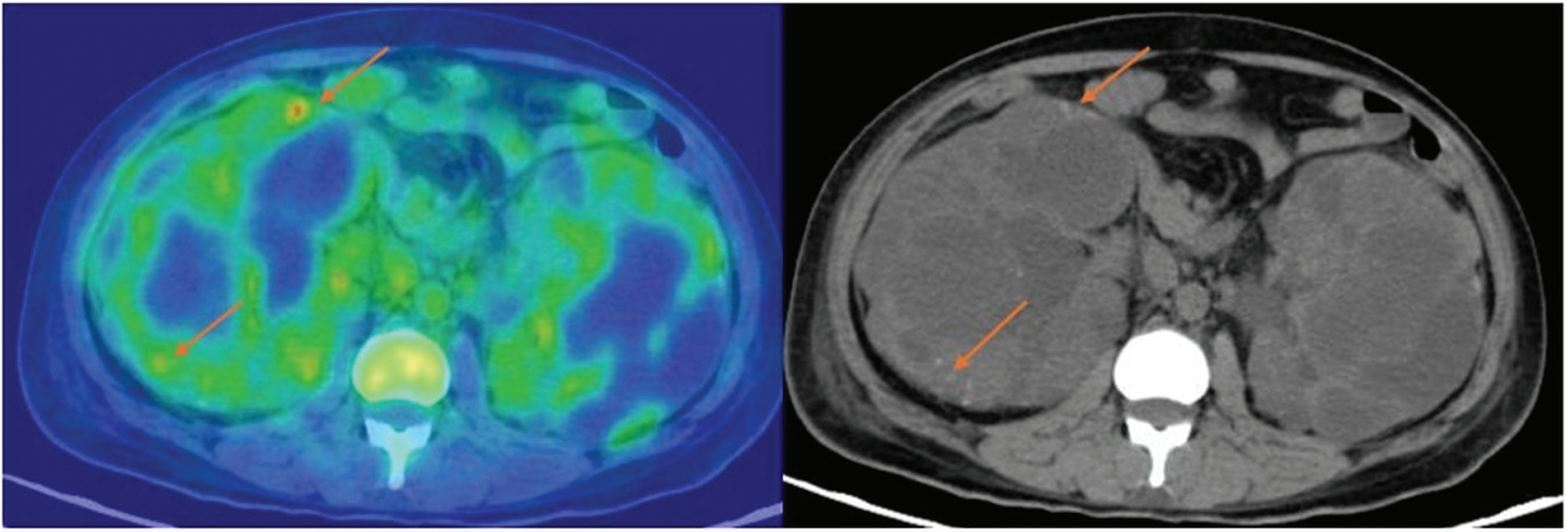
A 34-year-old gentleman with autosomal dominant polycystic kidney disease (ADPKD) and persistent fever, a Ga-68 citrate positron emission tomography/computed tomography (PET/CT) scan was performed to rule out infection or hemorrhage, Ga-68 citrate avidity was noted in areas corresponding to hyperdense cysts in both the kidneys and was further advised for ultrasonography (USG)-guided aspiration from the target lesions for further confirmation, which was performed and confirmed as hemorrhage with no infective growth in the culture. This was regarded as a false positive finding.

Five patients were evaluated for soft tissue-related infections. Three had a negative scan, and the findings were confirmed with a pus culture. Two patients had a positive scan; one patient had a positive pus culture and the other had a negative one, which was a false positive scan finding (clinical details in Table 3 and Fig. 3 ). The patient who had a false positive scan was a gentleman with ADPKD and persistent fever, a Ga-68 citrate PET/CT scan was performed to rule out infection or hemorrhage, Ga-68 citrate avidity was noted in areas corresponding to hyperdense cysts in both the kidneys and was further advised for USG-guided aspiration from the target lesions for further confirmation, which was performed and confirmed as hemorrhage with no infective growth in the culture. This was regarded as a false positive finding.

**Table 3:** False positive patient details ( Fig. 3 )

Patient profile	35 y, M
History	ADPKD (autosomal dominant polycystic kidney disease) and persistent fever, dysuria, flank pain for 14 days, to rule out infection or hemorrhage in the renal cyst
Nonenhanced CT scan findings	Nonenhanced CT scan showed bilateral enlarged kidneys with multiple hyperdense cysts in both the kidneys—which was likely hemorrhagic in nature
Ga-68 citrate PET/CT scan findings	Ga-68 citrate imaging was done to rule out hemorrhagic cyst versus and infected cyst and showed multiple cysts with Ga-68 citrate avidity in bilateral kidneys, which were hyperdense on the CT images
USG-guided FNAB findings	A USG-guided FNAB was performed, and the hemorrhagic aspirate was sent for culture, which was negative for any infective growth
Biochemical parameters	CRP: 155 mg/L WBC: 14,500/cumm

Abbreviations: CT, computed tomography; CRP, C-reactive protein; FNAB, fine-needle aspiration biopsy; PET, positron emission tomography; USG, ultrasonography.

### Semiquantitative Assessment

Semiquantitative assessment with SUVmax from the region of interest was retrospectively assessed. The SUVmax was compared with the opposite normal side soft tissue/bone or the blood pool uptake. A positive Ga-68 citrate PET/CT scans presented with a mean SUVmax of 7.79 ± 3.58 (range: 3.38–17.98) for a confidence level of 95%. A negative Ga-68 citrate PET/CT scans presented with a mean SUVmax of 2.65 ± 0.48 (range: 1.60–3.48). The overall population, including both positive and negative groups, presented with a mean SUVmax of 6.59 ± 1.348 (range: 1.60–17.98) for a confidence level of 95%.

### Follow-Up Patient Results

**Fig. 4:**
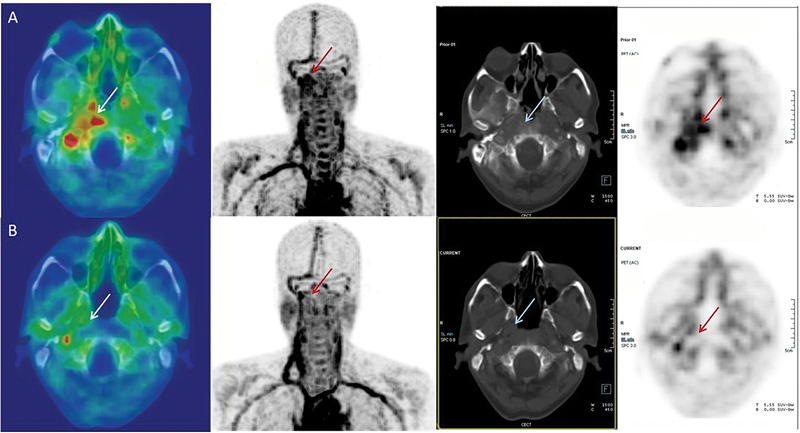
A 62-year-old gentleman had history of left skull base osteomyelitis (SBO) and underwent left radical mastoidectomy prior to his visit at our hospital. His presenting complaints included right-sided otalgia associated with progressive hearing loss. ( **A** ) Ga-68 citrate positron emission tomography/computed tomography (PET/CT) done showed citrate avid heterogeneously enhancing thickening in the right nasopharynx and petro-occipital region with minimal citrate avidity in the areas of soft tissue thickening in the central and both lateral skull base regions. ( **B** ) The patient underwent surgical debridement and antibiotic therapy and a follow-up Ga-68 citrate PET/CT done after 3 months did not show any citrate avid findings and contrast-enhanced CT images showed near-complete resolution of the soft tissue thickening involving the central and both lateral skull base regions.

Eight patients with SBO had a follow-up scan to assess their treatment response ( Fig. 4 ). *Complete metabolic response* was defined by the absence of residual Ga-68 citrate activity in the region of prior infection as compared with the pretherapy Ga-68 citrate scan. *Incomplete treatment response* was defined as residual activity in the region of prior infection but with a decrease in the area involved. *Failure of treatment* was defined as an increase in Ga-68 citrate activity with new areas of activity/involvement.

Four patients (clinical details mentioned in Table 4 ) had good treatment responses, one with SBO and three with long bone chronic osteomyelitis. A good correlation was noted with inflammatory marker CRP, which had normalized during follow-up imaging. Three patients (clinical details mentioned in Table 5 : Sl. no. 1–3) with SBO who had undergone surgical debridement and antibiotic treatment had incomplete treatment responses. Two of them had an MRI, which showed evidence of residual infection. Inflammatory markers showed a declining trend but had not normalized during the time of follow-up imaging. One patient (clinical detail mentioned in Table 5 : Sl. no. 4) with left SBO had treatment failure, which was picked up on the follow-up scan and correlated with MRI findings and rising inflammatory markers. New areas of involvement were noted, and he was found to have bilateral SBO, indicating treatment failure with disease progression.

**Table 4:** Clinical details of patients with complete treatment metabolic response on follow-up imaging

Treatment response						
Sl. no.	Clinical detail	First Ga-68 citrate scan finding	Other parameters s/o infection	Treatment detail	Second Ga-68 citrate scan finding	Other parameters in agreement with scan findings
1.	SBO	Positive for SBO with extensions	Positive pus culture and biopsy, high ESR, and CRP, MRI s/o infection	Surgical debridement	Negative	CRP, TLC normalized
2.	Chronic osteomyelitis – right ankle	Positive for right ankle osteomyelitis, with associated soft tissue thickening and edema	Positive pus culture and biopsy, high ESR, CRP, and TLC	Medical management with antibiotics	Negative	ESR, CRP showed declining trend with normal TLC
3.	Chronic osteomyelitis – left femur	Positive for left femur osteomyelitis with diffuse cortical thickening and remodeling	Positive pus culture and biopsy, high ESR, CRP, and TLC	Surgical debridement with washout	Negative	ESR, CRP showed declining trend
4.	Chronic osteomyelitis – right distal femur ( Fig. 2 )	Positive for osteomyelitis in the right distal femur with fracture involving the distal metadiaphyseal region	Positive pus culture and biopsy, high ESR, CRP, and TLC	Surgical debridement with washout	Negative	ESR normalized

Abbreviations: CRP, C-reactive protein; ESR, erythrocyte sedimentation rate; MRI, magnetic resonance imaging; SBO, skull base osteomyelitis; s/o, suggestive of; TLC, total leukocyte count.

**Table 5:** Clinical details of follow-up patients with incomplete treatment response

Incomplete treatment response (Sl. no. 1–3) and disease progression (Sl. no. 4)							
Sl. no.	Clinical detail	Ga-68 citrate scan finding	Other parameters s/o infection	Treatment detail	Second Ga-68 citrate scan finding	Other associated parameters s/o residual infection	Further management
1.	SBO	Positive for SBO with involvement of retropharyngeal space	Positive pus culture and biopsy, high ESR, CRP, MRI with features s/o infection	Surgical debridement	Positive	MRI with persistent features, raised ESR, declining CRP and TLC	Antibiotics for residual disease
2.	SBO	Positive for right-sided SBO with involvement of right nasopharyngeal wall, right parapharyngeal space, right carotid space	Positive pus culture and biopsy, high ESR, high CRP, MRI with features of infection	Surgical debridement	Positive	MRI with persistent features, raised CRP, normal TLC	Antibiotics for residual disease
3.	SBO	Positive for left SBO involving the left parapharyngeal space	Positive biopsy, high CRP, MRI with features of infection	Surgical debridement	Positive	ESR, CRP showed declining trend with normal TLC	Antibiotics for residual disease
4.	SBO	Positive for left SBO involving left lateral wall of the nasopharynx	Positive pus culture and biopsy, high ESR and CRP, MRI with features of infection	Surgical debridement	Positive	CRP and TLC increased, MRI with persistent features	Antibiotics for disease progression

Abbreviations: CRP, C-reactive protein; ESR, erythrocyte sedimentation rate; MRI, magnetic resonance imaging; SBO, skull base osteomyelitis; s/o, suggestive of; TLC, total leukocyte count.

Ga-68 citrate could localize in a broad spectrum of bacteria, including both Gram-positive and Gram-negative (details in Table 6 ). Two species of fungi, that is, *Aspergillus fumigatus* and *Candida albicans* , were also noted in tissue diagnosis of patients with a positive Ga-68 citrate PET/CT scan (details in Table 6 ).

**Table 6:** Ga-68 citrate localizing organisms

Pathogen type	
Bacterial	Methicillin-resistant *Staphylococcus aureus*
	*Pseudomonas aeruginosa*
	Klebsiella species
	Streptococcus, β-haem. Group A
Fungal	*Aspergillus flavus*
	Candida

## Discussion

Among the limited studies on the utility of Ga-68 citrate in the diagnosis of infection, it has been best documented in the diagnosis of osteomyelitis. SBO 16 is a disease with significant morbidity and mortality risks and occurs mostly in patients with diabetes mellitus or with immunocompromised status. The two variants of SBO are lateral SBO and central SBO (CSBO). Patients present with unrelenting otalgia that is disproportionate to the clinical signs, persistent purulent otorrhea, granulation tissue at the bony cartilaginous junction of the external auditory canal, and facial nerve paralysis. CSBO patients present with persistent headache, with nocturnal increase in intensity. Both varieties of SBO lead to eventual development of lower cranial nerve paralysis and vascular thrombosis. It is crucial for the surgeon to identify suitable sites for biopsy and debridement, as identification of the pathogen guides the clinician to initiate appropriate antimicrobial therapy. As per literature 17 there is usually a delay of 70 days in diagnosis of SBO. In addition, the decision on its continuation/cessation to prevent relapse is equally important and challenging. MRI studies may be useful, but may be limited in identifying the active disease, as it solely relies on anatomical changes.

In our study, 81% of the SBO patients had tissue diagnosis suggestive of infection. Our study found good utility of Ga-68 citrate PET/CT in diagnosing SBO and in treatment response evaluation, which is often a challenge for the surgeon as a repeat biopsy is often invasive due to the proximity of critical structures. Treatment is most often surgical so a functional imaging modality like Ga-68 citrate with no physiological brain activity can be used as a reliable guide to plan surgical extent and assess intracranial involvement; this is a known drawback of F-18 FDG as a functional tracer in SBO as it has high physiological uptake in the brain. It also gives confidence to the treating physician on whether to continue or discontinue long-term antibiotics based on imaging findings.

Diagnostic biopsy of long bone osteomyelitis and prosthesis-related infections are not always successful with a sensitivity of 50%. 18 19 MRI is a sensitive modality but is often limited in the setting of early infections, postoperative edema, and metallic implants. 12 F-18 FDG, a commonly used tracer for malignancies, often shows falsely high uptake at prosthetic implant sites due to adverse reactions from the metallic debris. 20 F-18 FDG can also show falsely FDG uptake in regions of periprosthetic seroma, 21 adding to the nonspecificity. Tseng et al 15 compared the utility of Ga-68 citrate PET/CT and F-18 FDG PET/CT in the diagnosis of lower limb prosthesis-related infections and found Ga-68 citrate PET/CT had a higher specificity (88% vs. 38%) and promising results in being able to differentiate between aseptic inflammation from an infective focus. Our study found that Ga-68 citrate PET/CT had a specificity of 87.5%, which is similar to that of the abovementioned study by Tseng et al. Such a high specificity makes Ga-68 citrate a promising tracer for differentiating infection from aseptic inflammation. In our study, patients who were evaluated for long bone osteomyelitis, had good correlation between the scan findings and tissue diagnosis (75% true positives) and the rest had raised inflammatory markers. Among those with negative scan findings 40% were true negatives and the rest were likely negative due to normalized CRP level, a statistically significant marker of infection in this study. The criteria for interpreting prosthesis-related infection were similar to those used in the study by Tseng et al 15 in which specific uptake of the radiotracer in the bone prosthesis interface and radiotracer uptake around the prosthetic soft tissue, rather than the uptake intensity was considered positive.

The utility of Ga-68 citrate PET/CT in diagnosing soft tissue infections is challenging due to the high blood pool uptake, this was noted in our patient population wherein one patient had a false positive scan ( Fig. 3 ). A small cohort study 22 compared the utility of Ga-68 citrate PET/CT with F-18 FDG PET/CT for diagnosing soft tissue infection and found that F-18 FDG was superior in diagnosing soft tissue infections and was attributed to the high blood pool activity of Ga-68 citrate and its limited utility in delayed imaging due to the short half-life of the tracer. The utility of Ga-68 citrate in the diagnosis of brain infection, especially in postsurgical cases where symptoms might persist due to either residual infection/postoperative edema should be further explored since this tracer does not have physiological accumulation in the brain unlike F-18 FDG. A case study 23 reported a patient with tuberculous granuloma of the central nervous system and explained the utility of the tracer in diagnosing brain infection due to the lack of physiological biodistribution in the brain.

**Fig. 5:**
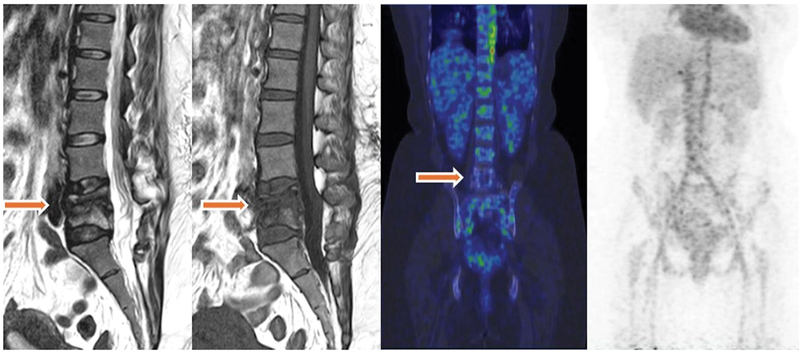
A 33-year-old lady with history of spinal tuberculosis involving L4-L5 vertebrae, came for end-of-treatment response after 2 years of antitubercular therapy (ATT) for spinal tuberculosis was symptom free. A Ga-68 positron emission tomography/computed tomography (PET/CT) citrate scan was performed to decide whether to stop ATT. The patient initially underwent a magnetic resonance imaging (MRI) scan for residual inflammatory changes and residual edema involving the L4-L5 vertebral bodies. A Ga-68 citrate PET/CT scan was performed 2 days later, which showed no tracer uptake in the previously involved region.

End-of-treatment response after 2 years of antitubercular therapy (ATT) for spinal tuberculosis was assessed in one patient ( Fig. 5 ). The patient was symptom-free and a Ga-68 citrate scan was performed to decide whether to stop ATT. The patient initially underwent an MRI scan, which showed residual inflammatory changes and residual edema involving the L4-L5 vertebral bodies. A Ga-68 citrate PET/CT scan was performed 2 days later, which showed no tracer uptake in the previously involved region, which enhanced the clinician's confidence in decision-making to stop ATT. A study done by Vorster et al 24 studied 13 patients who were previously diagnosed with tuberculosis and underwent a Ga-68 citrate PET/CT to look at the utility of Ga-68 citrate PET/CT in the detection of extrapulmonary sites, bone being one of the sites. Other sites included the lymph nodes, pleura, spleen, and gastrointestinal tract. Eighty percent more lesions were detected on Ga-68 citrate PET than on CT. Treatment response assessment evaluation done in eight patients showed good correlation with Ga-68 scan findings, biopsy, and CRP levels. It helped in guiding patient management plans and repeating biopsies from specific regions of highest uptake for culture-sensitive antibiotic therapy planning in refractory cases of SBO. Ga-68 citrate is a versatile tool for diagnosing a variety of pathogens and can contribute significantly to the need for specific diagnosis in this era of antimicrobial resistance.

We initially performed a 30-minute imaging protocol for the first 15 patients, sufficient to visualize the disease process, but there was high blood pool activity. Delayed imaging of 90 minutes was additionally done for one patient, which showed minimal degradation of image quality. Following this, a 60-minute protocol was performed and was sufficient to visualize the pathological process, giving enough time to improve the background-to-target ratio due to blood pool clearance, hence a 60-minute protocol was finalized. Several studies have looked at various uptake times and have noted that Ga-68 citrate can detect lesions within 30 minutes, and after that, an increase in the intensity of uptake in the lesions by 60 minutes due to blood pool clearance. 25 Another study 26 noted that the quality of images significantly deteriorated by 120 minutes.

## Limitations of the Study

We could not acquire delayed images with Ga-68 citrate due to the short half-life of the tracer. The radionuclide has less sensitivity for soft tissue infection due to the high blood pool activity of the tracer. The other limitations were small sample size, single-center experience, and interobserver variability in the scan interpretation. A comparison with F-18 FDG PET/CT was not performed; hence, we cannot comment on the additional advantages of one over the other.

## Conclusion

In conclusion, Ga-68 citrate is a promising tool for assessing the presence of infections, especially skeletal for diagnosis and treatment response assessment, significantly impacting clinical decision-making in accurately treating patients, especially useful in SBO patients, where planning the extent of surgery and decision on continuation of antibiotic treatment is challenging because clinical signs often persist despite adequate treatment response.
